# Polyamine and Ethanolamine Metabolism in Bacteria as an Important Component of Nitrogen Assimilation for Survival and Pathogenicity

**DOI:** 10.3390/medsci10030040

**Published:** 2022-07-29

**Authors:** Sergii Krysenko, Wolfgang Wohlleben

**Affiliations:** 1 Interfaculty Institute of Microbiology and Infection Medicine Tübingen (IMIT), Department of Microbiology and Biotechnology, University of Tübingen, Auf der Morgenstelle 28, 72076 Tübingen, Germany; sergii.krysenko@uni-tuebingen.de; 2 Cluster of Excellence ‘Controlling Microbes to Fight Infections’, University of Tübingen, 72076 Tübingen, Germany

**Keywords:** nitrogen assimilation, ethanolamine metabolism, polyamine metabolism, bacteria, drug discovery

## Abstract

Nitrogen is an essential element required for bacterial growth. It serves as a building block for the biosynthesis of macromolecules and provides precursors for secondary metabolites. Bacteria have developed the ability to use various nitrogen sources and possess two enzyme systems for nitrogen assimilation involving glutamine synthetase/glutamate synthase and glutamate dehydrogenase. Microorganisms living in habitats with changeable availability of nutrients have developed strategies to survive under nitrogen limitation. One adaptation is the ability to acquire nitrogen from alternative sources including the polyamines putrescine, cadaverine, spermidine and spermine, as well as the monoamine ethanolamine. Bacterial polyamine and monoamine metabolism is not only important under low nitrogen availability, but it is also required to survive under high concentrations of these compounds. Such conditions can occur in diverse habitats such as soil, plant tissues and human cells. Strategies of pathogenic and non-pathogenic bacteria to survive in the presence of poly- and monoamines offer the possibility to combat pathogens by using their capability to metabolize polyamines as an antibiotic drug target. This work aims to summarize the knowledge on poly- and monoamine metabolism in bacteria and its role in nitrogen metabolism.

## 1. Nitrogen Metabolism

### 1.1. Fundamentals of Nitrogen Assimilation in Prokaryotes

Nitrogen belongs to the group of vital elements. It is a key macronutrient required for the growth of living organisms. It is an essential component of amino acids, amino sugars, coenzymes, purines and pirimidines in nucleic acids [1,2], polyamines, and monoamines [3]. Microorganisms can use nitrogen sources with different redox states, including molecular nitrogen (redox state 0), ammonium (redox state −3), nitrate (redox state +5), and nitrite (redox state +3). Energetically, ammonium is the most preferred nitrogen source as a compound in the most reduced form.

Pathways for nitrogen assimilation comprise utilization pathways from the extracellular environment and biosynthetic pathways for intracellular production. Depending on the organism, these pathways are coordinated in order to control the intracellular amounts of nitrogen-containing compounds. Depending on the intracellular pool of nitrogen, expression of enzymes of nitrogen metabolism must be well coordinated. Nitrogen control has previously been precisely investigated in Gram-negative bacteria *Escherichia coli*, *Salmonella typhimuium*, *Pseudomonas aeruginosa*, as well as Gram-positive actinobacterial species *Corynebacterium glutamicum*, *Streptomyces coelicolor*, and *Mycobacterium tuberculosis* [4,5,6,7,8].

Some nitrogen-containing compounds such as ammonium can enter the cell by diffusion. Other compounds, e.g., amino acids, nitrate, nitrite, and urea, can be taken up by transporters [9]. A favored inorganic nitrogen source is ammonium which can diffuse across cell membranes or be transported into the cells via specialized Amt transporters. Subsequently, it can be directly used in the synthesis of glutamine and glutamate, which are key building blocks for biomolecules and subsequent biomass generation. Other inorganic nitrogen sources including nitrate, nitrite, urea, and diatomic nitrogen need to be reduced to ammonium before they can be assimilated. This process requires extra energy—in the form of ATP and electrons [5]. Organic nitrogen sources such as amino acids and amino sugars can be incorporated into the metabolism directly or in the deaminated and deamidated forms [8,10,11,12,13]. Alternative complex organic and non-organic nitrogen sources including monoamines and polyamines first have to be neutralized and detoxified with subsequent reduction [14,15].

**Figure 1:**
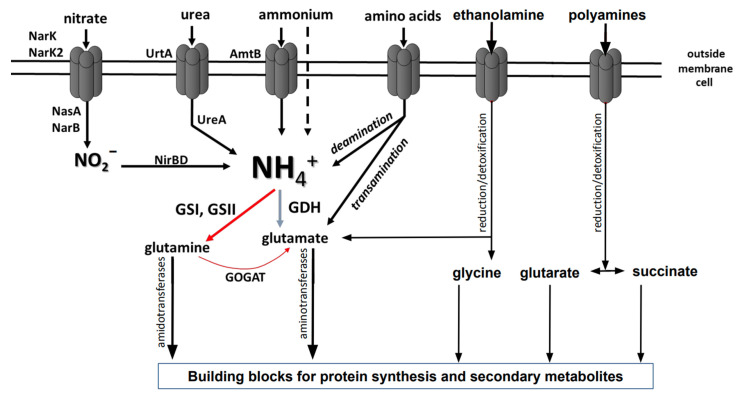
Schematic illustration of bacterial nitrogen metabolism. The main nitrogen sources, their uptake, and assimilation routes are depicted, as well as representative example of proteins involved in these processes. Enzyme systems for ammonia assimilation are shown in detail. GS: glutamine synthetase; GOGAT: glutamine-2-oxoglutarate-aminotransferase; GDH: glutamate dehydrogenase. Red arrows: GS/GOGAT pathway; light gray arrow: GDH pathway; black arrows: other metabolic routes; dark gray cylinders: transporters [5,6,16].

Bacteria possess two enzyme systems for ammonium assimilation based on the glutamine synthetase/glutamate synthase (GS/GOGAT) and glutamate dehydrogenase (GDH) activity, which are ubiquitous in bacteria. Under different nitrogen concentrations, these two distinctive pathways form glutamine and glutamate, respectively. The intracellular ammonium is integrated into cellular metabolism by the glutamine synthetase. It is active under low concentrations of ammonium as a component of the glutamine synthetase/glutamate synthase (GS/GOGAT) pathway. Glutamine synthetase (GS) catalyzes the ATP-dependent synthesis of glutamate and ammonia to glutamine. Glutamate is generated from glutamine and 2-oxyglutarate by glutamine-2-oxoglutarate-aminotransferase (GOGAT) [5,6,15]. Glutamate synthase (GOGAT) transfers the amide group from glutamine to 2-oxoglutarate (2-OG), producing two molecules of glutamate. Under high concentrations of ammonium, the glutamate dehydrogenase (GDH) is active. It catalyzes the synthesis of glutamate using substrates ammonium and 2-oxyglutarate as well as NADPH. However, the GDH remains inactive under limiting conditions due to the low substrate affinity and high Km value [6] (Figure 1). Glutamine and glutamate can be subsequently incorporated into diverse biosynthetic reactions in the cell. Glutamine provides nitrogen for the synthesis of aromatic compounds (purines, pyrimidines), amino acids (arginine, histidine, tryptophane, asparagine), amino sugars (glucosamine), and others (e.g., aminobenzoate). Glutamate is the donor of nitrogen in transamination reactions [16,17] (Figure 1).

### 1.2. Nitrogen Assimilation in Gram-Negative Bacteria

For survival in a competitive and stressful environment under nutrient limitation, bacteria have developed a complex metabolism and regulatory machinery that controls the amount of nitrogen in the cell at transcriptional and post-transcriptional levels. Nitrogen control has been extensively investigated predominantly in enteric bacteria from the Enterobacteriaceae family: *Escherichia coli*, *Salmonella typhimurium*, *Klebsiella pneumoniae*, and *Klebsiella aerogenes*. A key role has the central response regulator NtrC, which is coupled with a specific sensor kinase NtrB sensing the limitation of nitrogen. In addition, the status of nitrogen and the regulation of enzyme activity at a global level are controlled via the following proteins: encoded by *glnD* uridylyltransferase/uridylyl-removing enzyme UTase; an adenylylate transferase/deadenylylase GlnE; small signal transducing proteins PII GlnK and GlnB [6].

#### 1.2.1. Nitrogen Assimilation and Control in *Escherichia coli*

In *E. coli*, nitrogen starvation conditions induce the differential expression of genes that are regulated by the two-component system NtrB/NtrC. The PII signal protein GlnB is essential in this regulatory cascade sensing the cellular level of ATP, 2-oxoglutarate or glutamate [6]. The role of GlnK has also been demonstrated in *E. coli*. During nitrogen starvation, it regulates the expression of *Ntr* genes [18]. The PII protein can occur in two different forms in *E. coli*: PII and urydylylated PII-UMP. The bifunctional uridylyltransferase GlnD is responsible for two different states of the PII protein, whereas both activities are influenced by the amounts in the cell of the 2-oxoglutarate/glutamine and ATP/ADP. High carbon and ATP amounts signal the nitrogen limitation stimulating the activity of the uridylyltransferase. High concentrations of glutamine stimulate the uridylyl-removing activity of UTase. Under nitrogen-limiting conditions, urydylylated PII protein senses the phosphorylation of the sensor kinase NtrB [6]. Phosphorylated NtrB can activate the response regulator NtrC via the transfer of the phospho-group [19]. Phosphorylation of NtrC changes its conformation forming a dimer of two NtrC molecules. The dimeric form of NtrC enhances the transcription of nitrogen metabolism genes, including the gene *glnA* encoding glutamine synthetase through binding to the σ^54^ sigma factor-dependent promoters. Furthermore, in free-living anaerobic nitrogen-fixing bacteria such as *Rhizobia* sp. and *Klebsiella* sp., strict regulation of the *nif* genes for nitrogen fixation is present and depends on nitrogen availability. For example, in *Klebsiella pneumoniae*, the NtrB/NtrC system has been demonstrated to be required for control of the *nifL* gene for nitrogen fixation [20].

Nitrogen assimilation was also described to be controlled on the post-translational level. It occurs via covalent adenylylation and deadenylylation of each subunit of the glutamine synthetase enzyme [21,22]. This requires the bifunctional protein GlnE, which is regulated by the PII signal protein. In *E. coli*, modification of GSI by the covalent addition of AMP leads to a reduction of enzyme activity [23]. GlnE has two distinct domains, the N-terminal deadenylylation domain and the C-terminal adenylylation domain, that catalyze two different reactions. These domains are connected by an interregion linker. Besides the GS binding region, GlnE also has independent glutamine, PII, and PII-UMP binding sites. Under glutamine-rich conditions, glutamine and PII bind to GlnE. This causes the linker region to bring the adenylylation and deadenylylation domains together allowing adenylylation [23]. Under glutamine-limiting conditions, PII-UMP binds to GlnE. This causes a conformation change and allows deadenylylation [24].

#### 1.2.2. Nitrogen Assimilation and Control in Cyanobacteria

In cyanobacteria, nitrogen is catabolized via the coupled reaction in the GS/GOGAT pathway [25]. In contrast to other bacteria, in cyanobacteria, the GS activity is mainly regulated by small inhibitory proteins. Such proteins have been characterized in the *Synechocystis* PCC 6803 strain: inactivating factor 7 (IF7) encoded by *gifA* and inactivating factor 17 (IF17) encoded by *gifB* [26]. The global nitrogen control factor NtcA controls the expression of these genes responding to the 2-OG levels of the cell. There is no sensor protein interacting with NtcA. For the regulation of NtcA activity, PII is dispensable. Most cyanobacterial genomes harbor only one *glnB* gene, which encodes a PII protein. The others possess both *glnB* and *glnK* [27]. NtcA has been demonstrated to respond to 2-OG [28]. A global repressor of carbon-regulated genes, NdhR has been observed to use 2-OG as corepressor [29]. 2-OG appears as a main indicator of the status of central metabolism, carbon, and nitrogen catabolism. Furthermore, the regulation can be mediated by a non-coding RNA, a glutamine riboswitch in the 5′UTR of *gifB*, and by nsiR4. This glutamine riboswitch links the glutamine status of the cells to regulation of the central nitrogen assimilation reaction [27].

Several signaling pathways exist at different levels in cyanobacteria. Nitrogen-regulated genes *nirB* and *ntcB* have been characterized for example in *Synechococcus*, where they constitute an operon (*nirB-ntcB*) transcribed different from *nirA*. Under nitrogen limitation, transcription of *nirB*-*ntcB* is increased and is NtcA-dependent, whereas *ntcB* codes a member of the LysR family of transcriptional activators [6]. Cyanobacteria are able to maintain nitrogen fixation that can occur in cells heterocysts, which differentiate from the vegetative cell filaments. Nitrogen fixation as well as heterocyst development are controlled by ammonium repression. Ammonium represses expression of the nitrate reductase, nitrate/nitrite transport system, nitrite reductase, GSI, and the GSIII structural gene *glnN* [6,25,30].

### 1.3. Nitrogen Assimilation in Gram-Positive Bacteria

Gram positive bacteria are classified in two phyla: Firmicutes with low GC DNA content (subdivided into the classes Bacilli and Clostridia) and Actinobacteria with high GC DNA content (subdivided into multiple orders). The order *Actinomycetales* is the largest one, containing a variety of species and unclassified isolates. The nitrogen assimilatory enzymes of the GOGAT/GS pathway as well as GlnK and GlnE are conserved in Gram-positive bacteria such as *Clostridium acetobutylicum*, *Bacillus subtilis*, and *Streptomyces coelicolor*. However, while the enteric bacteria possess the Ntr control system, there is no reported presence of this system in Gram-positive bacteria. Instead, they possess alternative transcriptional regulatory mechanisms.

#### 1.3.1. Nitrogen Assimilation and Its Control in *Bacillus subtilis*

The model organism for Gram-positive bacteria with low GC content is *Bacillus subtilis*. Nitrogen metabolism has been extensively investigated in this organism. A preferred nitrogen source for this bacterium is ammonium [31]. No GdhA activity has been reported in *B. subtilis*—ammonium can be assimilated only via the GS/GOGAT pathway [32]. Furthermore, no post-translational modification of the GS enzyme has been observed, but feedback inhibition of GS by glutamine has been described. Under nitrogen depletion, three global regulators, GlnR, TnrA, and CodY, are present in cells for best possible growth [2]. Another regulator, GltC, controls the transcription of the glutamate synthetase (GOGAT) gene in dependence to the intracellular concentration of 2-oxoglutarate [33]. The transcriptional regulator TnrA that belongs to the MreR family of DNA-binding proteins has been shown to be functional only under nitrogen-limiting conditions in *B. subtilis*. The TnrA regulator positively controls the transcription of genes involved in ammonium uptake (*amtB*, *ureABC*, *nasBC*, and *nasDEF*) and nitrogen signaling (*glnK*), as well as its own transcription. In addition, it exerts a negative effect on the *glnRA* operon encoding GlnR and the GS, on the *gltAB* operon encoding GOGAT, and on *gltC* encoding GltC [34]. A homologue of the TnrA regulator, GlnR, can target almost the same binding sequences as TnrA. GlnR is active under nitrogen excess and acts mainly as a repressor. It represses the transcription of the *glnA* gene encoded in the *glnRA* operon as well as the transcription of *tnrA*, *gltAB*, *ureABC*, and *glnR* [35,36]. In *B. subtilis*, CodY-dependent regulation occurs under carbon and nitrogen depletion conditions when bacteria have to rely on amino acids for growth. The repressor protein CodY controls the transcription of urease encoding operon *ureABC*, dipeptide degradative operon *dpp*, histidine degradative operon *hut*, and isoleucine/valine degradative operon *bkd* [34].

#### 1.3.2. Nitrogen Assimilation and Its Control in *Corynebacterium glutamicum*

The model organism for Gram-positive bacteria with high GC content is *Corynebacterium glutamicum*, which has been extensively studied because of its application in industry for the production of amino acids such as L-glutamine. *C. glutamicum* also serves for studies of important human pathogens including *Corynebacterium diphteriae*, *Corynebacterium jeikeium*, *Mycobacterium leprae*, and *Mycobacterium tuberculosis*. *C. glutamicum* possesses the genes *amtA* and *amtB* for ammonium uptake [37], which are transcribed under nitrogen starvation conditions [38]. AmtA specifically transports methylammonium; AmtB transports ammonium with high affinity and methylammonium with low affinity [39,40]. In *C. glutamicum*, *amtB* forms a cluster with *glnK* coding for the signaling protein PII. Besides the nitrogen-sensing function, GlnK has reportedly been involved in the transcriptional regulation of nitrogen metabolism genes in combination with the global transcriptional regulator AmtR [41]. In *C. glutamicum*, ammonium is assimilated via the GS/GOGAT pathway under nitrogen limitation or via the Gdh pathway under nitrogen excess. Two GS enzymes have been described in *C. glutamicum*: GlnA (also annotated as GlnA1, GSI) and GlnA2 (GSI). GlnA is the essential functional glutamine synthetase which is subjected to post-translational modification by the adenylyl transferase enzyme GlnE [42,43]. GlnA2 has been described as a non-essential enzyme which is not subjected to post-translational modifications. In contrast to *E. coli*, GlnE activity in *C. glutamicum* is not regulated by GlnK [44]. A central transcriptional regulator of nitrogen metabolism genes in *C. glutamicum* is AmtR, which belongs to the TetR family of regulatory proteins typically acting as transcriptional repressors. The GlnK protein can interact with AmtR under nitrogen-limiting conditions. As a result of the protein–protein interaction, AmtR is removed from the upstream regions of its target genes and leaves them for transcription. AmtR has been reported to control its own transcription as well as the transcription of 34 other target genes including the *amtB–glnK–glnD* operon, the *gltBD*, *urtABCDE*, *gluABCD* operons, genes *dapD*, *gdh*, *codA*, the *ureABCEFGD* operon, genes *amtA*, *glnA1*, and *crnT* [40,45].

#### 1.3.3. Nitrogen Assimilation and Its Control in *Streptomyces coelicolor*

Intensive investigations of primary and secondary metabolism in *S. coelicolor* A3 (2) lead to establishment of this Gram-positive bacterium with high GC content as a model organism for *Streptomyces* and Actinobacteria [46]. In its natural soil habitat, *S. coelicolor* is an obligatory aerobe bacterium with filamentous growth and GC content of 63–78%. It belongs to the phylum Actinobacteria and genus *Streptomyces*, and is closely related to human pathogens from genus *Mycobacterium* and *Rhodococcus* [47,48,49]. Streptomycetes including *S. coelicolor* feature an impressive adaptability to environmental stress, a complex metabolism and life cycle, and high metabolic potential, as well as the ability to synthesize a large variety of useful natural products. *Streptomyces* spp. fulfill a key ecological role in the soil—they are able to naturally recycle the remains of other organisms by their utilization [50].

In the natural soil environment, *S. coelicolor* lives under varying nutrient conditions. Therefore, it is capable of assimilation of different sources of carbon and nitrogen such as ammonium (NH^4+^), nitrite (NO^2−^), nitrate (NO^3−^), amino acids histidine and arginine, amino sugars, peptides, urea as well as chitin, cellulose and xylose, releasing chitinases, cellulases, and xylanases for extracellular utilization of such organic material [51,52,53]. The genome of the *S. coelicolor* M145 strain was the first to be fully sequenced and is remarkably large: a linear chromosome contains 8,667,507 base pairs and 7825 predicted genes [49]. Diverse multiple gene clusters are present in the genome of *S. coelicolor* for the synthesis of natural products, such as actinorhodin, undecylprodigiosin, methylenomycin, perimycin, and calcium-dependent antibiotic [53]. Regulatory networks that control the metabolism and morphological differentiation of *S. coelicolor* in respond to nitrogen availability signals allow *S. coelicolor* to survive under variable nutrient conditions [54].

*S. coelicolor* is constantly exposed to environmental stress. This occurs in the form of limited availability of nutrient sources and local excess of toxic compounds as a result of the decomposition of organic material. The cellular response to nitrogen limitation in *S. coelicolor* can be controlled on a transcriptional level—it involves the global nitrogen response regulator GlnR [55,56]. It belongs to the OmpR family of regulators and influences by activation the expression of the operon *amtB*-*glnK*-*glnD* (the ammonium transporter AmtB, the PII signal protein GlnK, and the adenylyl transferase GlnD) that are conserved genes in *Actinomycetales* [57,58,59,60,61,62]. Other GlnR target genes include glutamine synthetase encoding genes *glnA* and *glnII*, a glutamate dehydrogenase encoding gene *gdhA*, nitrate/nitrite reduction genes *nirB* and *nasA*, urea cleavage gene *ureA*, and a HemD-like transcriptional regulator encoding gene *nnaR*, as well as seven further genes with an unknown function [57,58,61,63,64].

Two transcriptional regulators, GlnR and NnaR, control nitrate assimilatory genes (*nirBD*, *narK*, *nasA*) in *S. coelicolor*. Under nitrogen-limited conditions, GlnR can activate the expression of these genes that is enhanced by the synergistic binding of GlnR and NnaR in the presence of nitrate [65]. Another regulator, GlnRII, can bind to the upstream regions of *amtB*-*glnK*-*glnD*, *glnA*, and *glnII* as well as *sco1863* showing the same binding capacity as GlnR [60]. It was hypothesized that GlnRII has a particular role in the *glnII* regulation and is not a functional homologue of GlnR. It was found in *Streptomyces* spp., but has not been found in *Corynebacterium* and *Mycobacterium* [57,66].

Complex nitrogen metabolism of *S. coelicolor* requires additional control by further transcriptional regulators: Crp [67], PhoP [68,69], ArgR [70], AfsR [71], DasR [72,73], and AfsQ1 [74]. Crp regulates the interplay of primary and secondary metabolism, and the genes *amtB*-*glnK*-*glnD*, *glnA*, and *glnII* [67]. PhoP negatively regulates the transcription of the *amtB*-*glnK*-*glnD* operon as well as genes *glnA*, *glnII*, and *glnR* under conditions of phosphate limitation [26]. AfsR controls expression of *glnR* in response to unknown nutrient stress stimulus [71]. AfsQ1 is required for the carbon, nitrogen, and phosphate metabolism regulation under glutamate presence [74,75].

In dependence from the level of ammonium, the uptake in *S. coelicolor* can be controlled at post-translational level—the ammonium transporter AmtB interacts with the nitrogen sensor protein PII (GlnK) [57,76,77]. When concentrations of ammonium are high, GlnK can be inactivated by adenylylation mediated by the adenylyltransferase GlnD or by proteolysis [57]. In order to avoid the depletion of the intracellular glutamate pool, the activity levels of glutamine synthetases can be controlled at post-translational level [45]. When nitrogen conditions are variable, GlnA activity can be regulated through the reversible adenylylation/deadenylylation by an adenylyltransferase GlnE [60]. However, no post-translational modifications of the GOGAT enzyme in Actinobacteria have so far been reported. The regulation of GSI by GlnE has also been demonstrated in *E. coli*, where GlnE can be controlled by GlnK, GlnB (nitrogen regulatory protein P-II), and GlnD. However, in *S. coelicolor*, the PII proteins GlnK and GlnD seem to be not essential for these processes [45,77,78].

#### 1.3.4. Nitrogen Assimilation and Its Control in *Mycobacterium tuberculosis*

Nitrogen metabolism has been extensively studied in pathogenic Actinobacteria from the genus *Mycobacterium*, which includes soil bacteria such as *Mycobacterium smegmatis* as well as mammal and human pathogens including *Mycobacterium tuberculosis* (causative agent of tuberculosis) and *Mycobacterium leprae* (causative agent of leprosy). In *M. tuberculosis*, the glutamine synthetase and nitrogen assimilatory protein GlnA (also referred as GlnA1) have been associated with pathogenicity and virulence [79,80]. Understanding of nitrogen assimilation in this bacterium is important for the comprehension of infection mechanisms. Furthermore, this knowledge allows the development of novel therapeutic strategies to control *M. tuberculosis* and multidrug resistance (MDR) strains. *M. tuberculosis* possesses an ammonium transporter protein, AmtB, and it does not have an active glutamate dehydrogenase (GDH) enzyme. Thus, GS/GOGAT is the only way to assimilate nitrogen. *M. tuberculosis* genome contains one glutamine synthetase encoding gene *glnA* (also referred as *glnA1*) and three GS-like enzymes encoded by *glnA2*, *glnA3*, and *glnA4* [81,82]. All of these enzymes belong to the GSI (prokaryotic) type of glutamine synthetases. Although all GS-like enzymes have been reported to be active in cells, only GSI encoded by *glnA1* has been found to be essential for *M. tuberculosis* growth. The activity of the GlnA1 enzyme can be down-regulated under nitrogen excess by the bifunctional adenylyl transferase GlnE. Under nitrogen starvation, GlnE deadenylylates GlnA1 and restores its activity. In contrast to *E. coli* and similar to *S. coelicolor*, in *M. tuberculosis*, GlnE activity is not regulated by GlnK and GlnD [23]. At transcriptional level, GlnR, which is a functional homologue of the global transcriptional regulator GlnR from *S. coelicolor*, controls the nitrogen assimilation. As occurs in *S. coelicolor*, during nitrogen limitation in *M. tuberculosis*, GlnR regulates the transcription of *glnA* as well as the transcription of operons *amtB*-*glnK*-*glnD*, *gltBD*, and *nirBD*, and at least 33 other genes in *M. tuberculosis* and more than 100 genes in *M. smegmatis* [83,84]. A putative TetR-like transcriptional regulator, AmtR, has also been found in *M. tuberculosis* (Rv3160c) demonstrating only 27.9% amino acid sequence identity to the AmtR protein from *C. glutamicum* [45].

### 1.4. The Central Role of Glutamine Synthetases in the Bacterial Nitrogen Metabolism

Glutamine synthetases (GS; EC 6.1.1.3) are enzymes found in all forms of life, with the central role in nitrogen assimilation [85]. GS enzymes catalyze the formation of L-glutamine through the condensation of ammonia with L-glutamate in an ATP-dependent manner. Glutamine, together with glutamate, serves as an essential component for protein biosynthesis. It is a major nitrogen source for biosynthetic reactions in the cell and one of the major nontoxic ammonia carriers [86,87]. It has been assumed that glutamine synthetases might be among the most ancient existing enzymes in nature [88]. Functional GSs have been described to occur in three forms. GS type-1 (GSI) has been found in most prokaryotes [6,21,22] as well as in mammals and plants [89,90]. GSI enzymes are subdivided into two GS isoenzymes: GSI-α and GSI-β. GSI-α enzymes are generally found in thermophilic bacteria, low G + C Gram-positive bacteria, and euryarchaeota, whereas GS I-β enzymes are found in other bacteria [34]. GS type-2 (GSII) are generally found in eukaryotes, in some Gram-positive high GC-content bacteria [6,45,91], and in symbiotic Gram-negative soil bacteria such as *Rhizobium* sp. [88,92]. GS type-3 (GSIII) are generally found in cyanobacteria [93], in the Gram-negative anaerobe bacterium *Bacteriodes fragilis* [85], and some protozoans [94]. Another octamer form of GS, GSIV, has so far only been found in the plant-associated bacteria *Rhizobium leguminosarum* [95], *Rhizobium meliloti* [96], and *Agrobacterium tumefaciens* [97]. The biosynthetic activity of GSIV is significantly lower than that of GSI and GSII, which is the reason for discussions about whether the enzyme primarily fulfills a function other than the synthesis of glutamine [96]. However, this could not yet be demonstrated.

Structurally, all GSs are composed of two closed-ring structures. Active sites are formed between protomers [98]. GSI is a dodecameric protein with about 360 amino acids length and a subunit between 44–60 kDa that has been found in bacteria and archea [99,100,101,102]. GSII is a dodecamer with about 450 amino acids length, composed of subunits between 35–50 kDa [88,100,101,102]. GSIII is a hexameric enzyme with about 730 amino acids length, composed of subunits of approximately 75 kDa each [93,103].

The crystal structure of the glutamine synthases has been previously elucidated and characterized in *Salmonella typhimurium* [104,105], *Helicobacter pylori* [106], *Bacillus subtilis* [107], and *Mycobacterium tuberculosis* [108]. The GSI GlnA has been structurally elucidated in *S. typhimurium* (PDB: 2GLS). It consists of 12 subunits and demonstrates a high sequence similarity to prokaryotic GS enzymes [98,109]. The crystal structure of GSII (GlnII) with a resolution of 2.55 Å (PDB: 4BAX) has been determined in *S. coelicolor* and described by X-ray diffraction. GlnII is a dodecamer comprising 10 subunits organized in 2 rings that demonstrates high sequence similarity to eukaryotic GS enzymes [7,66,110]. The *glnII* gene has been detected in *Streptomyces* sp. but is not present in other Actinobacteria such as *Corynebacterium* and *Mycobacterium* [91] and is not transcribed at a constant level at all growth phases, though it is preferentially transcribed during mycelial differentiation [60]. The crystal structure of the GSIII (PDB: 3O6X) has been determined in *B. fragilis*—it is a dodecamer with two hexameric rings [103].

Furthermore, a computational analysis of *glnA*-genes across actinobacterial genomes revealed the existence of a supposed common *glnA* ancestor, from which the *glnA*, *glnII*, and *glnA*-like genes in different Actinobacteria are derived [88,111]. For example, in *S. coelicolor* and in *M. tuberculosis*, three genes were identified as *glnA*-like: *glnA2*, *glnA3*, and *glnA4* [57,112]. GlnA and GlnII were demonstrated to be functional glutamine synthetases, and *glnA2*, *glnA3*, and *glnA4* were shown to encode GS-like enzymes that do not exhibit a glutamine synthetase activity in both *S. coelicolor* [57,112,113] and *M. tuberculosis* [80].

In *S. coelicolor*, analysis of GlnA2, GlnA3, and GlnA4 revealed that they share similar structural features [7]. A phenotypic analysis of *glnA2*, *glnA3*, and *glnA4* knock-out mutants with different nitrogen sources demonstrated their involvement in polyamine and ethanolamine metabolism [113,114,115]. Biochemical studies on GlnA2 and GlnA3 demonstrated that they are active as γ-glutamylpolyamine synthetases [113,115]. Furthermore, it has been shown that GlnA4 is a γ-glutamylethanolamide synthetase [114]. The presence of different GS-like proteins highlights the occurrence of these specialized proteins required for the survival, colonization, and propagation in specific habitats.

## 2. Polyamine and monoamine Metabolism

### 2.1. Polyamine Metabolism in Bacteria

#### 2.1.1. Distribution and Role of Polyamines

Polyamines are aliphatic polycations that are composed of a polycarbone chain and amino groups. Widely distributed natural polyamines are putrescine (1,4-diaminobutane), spermidine (N-(3 aminopropyl)-1,4-butadiamine), spermine (N, N′-bis (3-aminopropyl-1,4-butanediamine)), and cadaverine (pentane-1,5-diamine) [116,117,118]. Further polyamines that are not frequently occurring in nature have been reported, including thermine, thermospermine, caldopentamine, and others. These molecules have been found in the extreme thermophile *Thermus thermophiles* [119]. A variety of other linear polyamines have been found in (hyper)thermophilic archea and bacteria, including norspermidine, norspermine, caldopentamine, homocaldopentamine, thermopentamine, and caldohexamine [120], as well as branched-chain polyamines such as N^4^-bis(aminopropyl)spermidine [121].

Polyamines are present in diverse bacterial habitats. In soil, the polyamine concentration per gram of humus ranges are: putrescine: between 0.28 and 0.56 nmol/g, spermidine: 0.23–0.62 nmol/g, and spermine: 0.16–0.43 nmol/g [122]. In mammal cells, a natural environment for pathogenic bacteria, elevated polyamine levels have been reported exceeding physiological concentrations in human skin, in regenerating tissues (more than 1 mM), and in body fluids and blood (more than 0.01 mM) [123,124,125]. In addition, polyamine levels increase in human lungs during an inflammation process and shortly after apoptosis [126]. However, increased polyamine concentrations can lead to bacterial cell death. For example, it has been reported that 0.15 mM of exogenous putrescine is toxic for the cyanobacterium *Anacystis nidulans* [127]. Other reports described inhibition of *E. coli* growth in the presence of 4 mM spermidine [128].

Natural intracellular polyamine concentrations range in different species. *E. coli* can synthesize high amounts of putrescine (up to 32 mM total concentration) and spermidine (up to 6.88 mM). However, in most bacteria, the content of intracellular putrescine ranges between 0.1–0.2 mM [3,129]. Spermidine has been shown to be essential for planktonic growth of some Gram-negative bacteria, such as ε-proteobacterium *Campylobacter jejuni* [130] and γ-proteobacterium *Pseudomonas aeruginosa* PAO1 [131]. The intracellular spermidine content can vary between 1–3 mM. In contrast to other polyamines, the presence of cadaverine and spermine in bacterial cells remains not well investigated. In *E. coli*, spermine is not biosynthesized de novo, but it can be taken up from the environment [132]. In other bacteria, spermine was found in cells when present in the medium [133]. Cadaverine has been characterized in bacteria and plants. It is the least prevalent of polyamines that naturally occur in cells and is of low importance for bacteria [134]. It is normally absent in *E. coli* [133].

Polyamines, especially spermidine, have been described as essential in Archaea and eukaryotes. However, their role in bacteria is less understood [13,118]. It has been reported that polyamines are not required for normal growth in Gram-positive bacteria such as *Bacillus subtilis* [135] and *S. coelicolor* [136]. In γ-proteobacteria, polyamines have been shown to be required for growth—their limitation reduced the growth rate in *Yersinia pestis* [137], *Vibrio cholera* [138], *Salmonella typhimurium* [139], and *Escherichia coli* [140]. The intracellular polyamine amount is tightly coordinated with the cellular metabolism. Polyamines have been demonstrated to be accumulated intracellularly under stress conditions [117]. In bacteria, polyamines are important for homeostasis: their functions include an influence on transcription [140,141,142,143] and translation [144,145,146,147], the biosynthesis of siderophores [136,148,149,150], cell growth stimulation [151,152], and biofilm formation [138,153,154]. Furthermore, polyamines play a role in stress response: they confer the response to oxidative stress [128,140,155,156], SOS system activation [157], acid resistance [155,158,159], and antibiotic resistance [160,161,162,163].

#### 2.1.2. Importance of Polyamines for Intracellular Pathogens

In mouse macrophages, the concentration of polyamines putrescine, spermidine, and spermine reportedly vary from 250–1750 pmol/5 × 10^6^ macrophages, depending on the metabolic state of the cell [164]. Polyamines synthesized by the host can favor intracellular survival of human pathogens causing deadly diseases. Such bacteria are able to colonize and manipulate immune cells, escaping the response. This makes the treatment of infections extremely difficult. Macrophages represent the first line of human immune defense. Most intracellular pathogens residing in macrophages cause a time-dependent up-regulation of the metabolic regulator (PPARγ) in infected macrophages, resulting in increased expression of M2 markers and down-modulation of the M1 response [165]. PPARγ induces the arginine metabolism leading to the synthesis of the polyamine spermine from putrescine via spermidine [166]. Bacteria that are able to colonize and survive in macrophages, providing access to intracellular polyamines, include *Klebsiella pneumonia* causing pneumonia infection [167], *Salmonella typhimurium* causing typhoid fever [168], *Brucella abortus* causing brucellosis [169], *Acinetobacter baumannii* causing meningitis or lung infections [170], and *Mycobacterium tuberculosis* causing tuberculosis [171].

#### 2.1.3. Occurrence of Polyamines in Bacterial Cells

##### Polyamine Biosynthesis

In bacteria and in all kingdoms of life, polyamines can be synthesized from the amino acids methionine, ornithine, lysine, and arginine [117,172]. Bacterial polyamine biosynthetic pathways are configured in multiple pathways that have been studied in *E. coli*, *P. aeruginosa*, *Salmonella enterica*, *Campylobacter jejuni*, and *S. coelicolor* [118]. Generally, bacteria produce a diamine (putrescine or cadaverine) and triamine (spermidine). Some bacteria can produce longer-chain polyamines (including spermine), some produce only diamines, and others, such as pathogens, often do not produce any polyamines [173].

**Figure 2:**
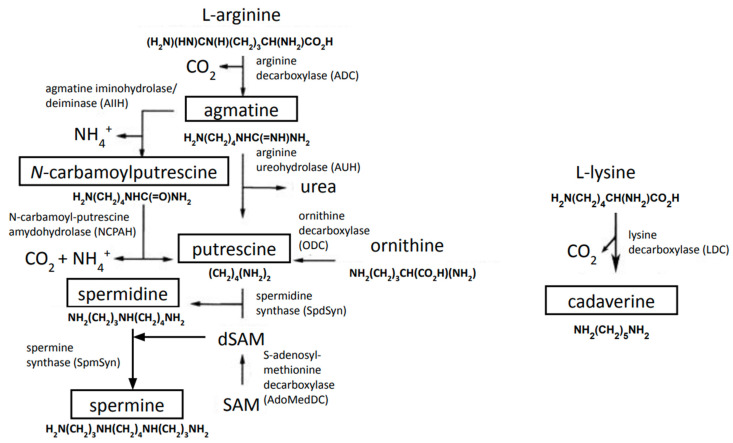
Combined polyamine biosynthetic pathways in bacteria. Enzymes that catalyze central reactions of polyamine biosynthesis as well as substrates and products of reactions are shown. Both ODC and ADC pathways and also LDC-dependent cadaverine production are depicted. Polyamine products are marked in boxes. SAM: S-adenosylmethionine; dSAM: decarboxylated S-adenosylmethionine [14,117].

Putrescine is synthesized by ornithine decarboxylation involving an ornithine decarboxylase (ODC). Furthermore, putrescine can be generated from arginine by an arginine decarboxylase (ADC) and agmatinase. Both ODC and ADC pathways have been extensively investigated in *E. coli* [117,174]. In *P. aeruginosa*, agmatine conversion to putrescine in a two-step reaction has been demonstrated. It involves hydrolytic deimination of agmatine to N-carbamoylputrescine catalyzed by agmatine deiminase AguA. Subsequently, N-carbamoylputrescine amidohydrolase AguB catalyzes hydrolysis of the carbamoyl group yielding ammonia, carbon dioxide, and putrescine [131,175]. The second reaction has also been shown in *Enterococcus faecalis*, where putrescine transcarbamylase releases carbamoylphosphate from N-carbamoylputrescine and inorganic phosphate yielding putrescine [176]. Spermidine and spermine are derived from putrescine via addition of aminopropyl groups, which are supplied by the methionine derivative—decarboxylated S-adenosylmethionine (dcAdoMet, dSAM) produced by a S-adenosylmethionine decarboxylase (AdoMetDC, SpeD). The addition of aminopropyl groups occurs through spermidine and spermine synthetases (SpdS/SpeE and SpmS) [117,174,177]. Cadaverine has been studied in *E. coli*, *Lactobacillus* spp. and *Vibrio* sp., where it is synthesized by lysine decarboxylases (LDCs)-dependent lysine decarboxylation [178] (Figure 2). In *S. coelicolor*, low amounts of putrescine, spermidine, spermine, and cadaverine (ca. 0.05–0.1 μmol/g) have been detected when grown in a complex medium [120]. It has been reported that putrescine, spermidine, and diaminopropan can occur in *S. coelicolor* in the late-stationary phase in the minimal liquid medium (NMMP), while cadaverine can be produced under iron limitation [136], hinting towards a possibility to synthesize these polyamines de novo in *S. coelicolor*. Knowledge of polyamine biosynthetic pathways have allowed advances in engineering of the industrial bacterium *C. glutamicum* for efficient production of the most promising polyamines putrescine and cadaverine [179].

##### Polyamine Uptake

On the one hand, some bacteria do not possess a polyamine biosynthetic pathway and must import polyamines from the extracellular environment. On the other hand, the uptake of polyamines is generally economical for bacteria and allows energy to be saved, because the synthesis of S-adenosylmethionine (AdoMet, SAM), for which biosynthesis requires ATP, is consumed in the AdoMet decarboxylase–spermidine synthetase pathway. The uptake of external polyamines is of central importance in order to optimize growth, metabolism, cell-to-cell communication, and adaptation to the environment [14].

At physiological pH, polyamines are positively charged hydrophilic molecules. They cannot pass through cellular membranes by diffusion. Thus, an active transport system is needed for polyamine uptake from the extracellular environment. Polyamine transport has been extensively investigated in predominantly pathogenic bacteria, including *Vibrio cholerae*, *Proteus mirabilis*, *Aggregatibacter actinomycetemcomitans*, *Streptococcus pneumonia*, and *Escherichia coli*.

**Figure 3:**
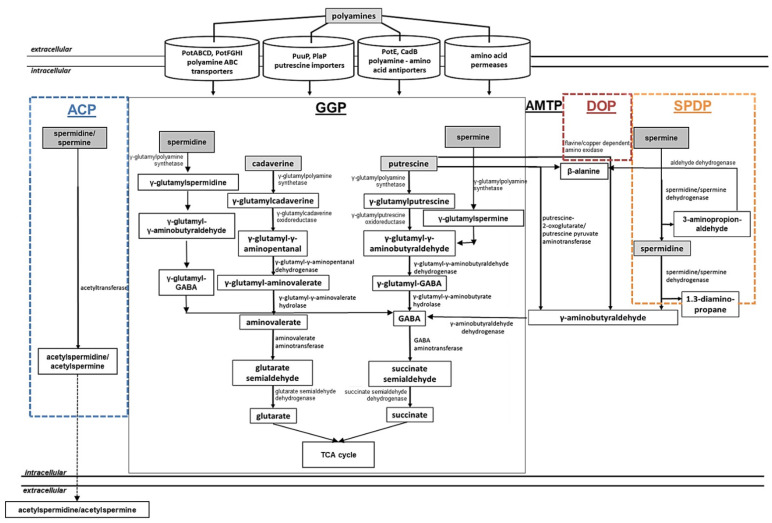
Combined model of bacterial polyamine utilization pathways (adapted from [7,115]). Central reactions and metabolic routes of polyamine catabolic pathways, as well as up-to-date polyamine uptake systems described in bacteria, are shown. ACP, acetylation pathway; GGP, gamma-glutamylation pathways (black box); AMTP, aminotransferase pathway; DOP, direct oxidation pathway; SPDP, spermine/spermidine dehydrogenase pathway. Dashed arrows represent predicted and straight arrows confirmed metabolic pathways. Pathways described for the following bacteria—in black: *E. coli*, *P. aeruginosa*, and *S. coelicolor*; dashed orange box: *P. aeruginosa*; dashed brown box: *S. aureus* and *P. aeruginosa*; dashed blue box: *E. coli*, *B. subtilis*, and *C. glutamicum* [7,14,195,197,198,201,206,207,208,212,215,217,218,219].

Several polyamine transporters have been described in *E. coli*: PuuP for putrescine [180,181], the putrescine–ornithine antiporter PotE [182], PotFGHI for putrescine [183], the proton-dependent importer PlaP for putrescine [184], the cadaverine–lysine antiporter CadB [185], the importer PotABCD for spermidine [186,187], and the spermidine transporter MdtJI [188] (Figure 3; Table 1). In *V. cholerae*, three homologues of PotD from *E. coli* have been described: NspS, PotD1, and PotD2 [189].

Polyamine uptake has been shown to influence the pathogenicity. The periplasmic substrate-binding protein PotD1 has been shown to be responsible for spermidine uptake that hinders biofilm formation. Instead, a polyamine norspermidine enhances biofilm formation via the NspS/MbaA signaling system in *V. cholerae* [190]. In *A. actinomycetemcomitans*, a homolog of PotD from *E. coli* was also reported to correlate with biofilm formation causing periodontitis [191]. In *S. pneumonia*, the uptake of spermidine by the spermidine importer PotABCD was reported to be important for pathogenicity in mice [133,192]. In *P. mirabilis*, putrescine uptake was demonstrated to be carried out by the proton-dependent putrescine importer PlaP [193].

#### 2.1.4. Polyamine Assimilation in Bacteria

Rather low amounts of polyamines can be synthesized intracellularly for maintenance of cellular functions. However, in the extracellular environment, polyamines can be present in excess, resulting in locally elevated and toxic amounts of these compounds. Under these conditions, detoxification of increasing concentrations of intracellular polyamines is required to avoid cell death. On the other hand, some bacteria do not have a complete pathway for polyamine biosynthesis (*Enterococcus faecalis*) or lack it completely (*Staphylococcus aureus*), thus relying on polyamine uptake and utilization [194]. It has been reported that bacteria can utilize polyamines as a source of nitrogen and carbon to control the intracellular polyamine pool. This process has been investigated in the Gram-negative bacteria *E. coli* and *P. aeruginosa* as well as in the Gram-positive bacteria *Staphylococcus aureus*, *Bacillus subtilis*, *C. glutamicum*, and *S. coelicolor* [7,14,15,180,195,196,197,198]. The following pathways have been characterized in *E. coli*, *P. aeruginosa*, *B. subtilis*, and *S. coelicolor*: the gamma-glutamylation pathways (Figure 3, GGP), the aminotransferase pathway (Figure 3, AMTP), the direct oxidation pathway (Figure 3, DOP), the spermine/spermidine dehydrogenase pathway (Figure 3, SPDP), and the acetylation pathway (Figure 3, ACP).

##### Polyamine Utilization in *E. coli*

The gamma-glutamylation pathway (also referred as the putrescine utilization pathway) has been characterized in *E. coli* and *P. aeruginosa* [180,199,200,201] (Figure 3, GGP). In *E. coli*, extracellular putrescine can be transported into the cell by the PuuP transporter and afterwards glutamylated by the γ-glutamylputrescine synthetase (PuuA) in an ATP-dependent manner [180,200] (Table 1). In further steps, the pathway involves the γ-glutamylpolyamine oxidoreductase PuuB, the γ-aminobutyraldehyde dehydrogenase PuuC, the γ-glutamyl-GAΒA hydrolase PuuD, the GABA aminotransferase PuuE, and the succinate semialdehyde dehydrogenase leading to the production of succinate [199,202] (Figure 3, GGP; Table 1). A cadaverine-specific utilization pathway has not been reported in *E. coli*, but the activity of PuuA with cadaverine was comparable of that towards putrescine in vitro [14,203]. Presumably both the aminotransferase and the glutamylation pathway of putrescine are involved in cadaverine metabolism in *E. coli*.

The aminotransferase pathway has been investigated in *E. coli* [201,204,205] (Figure 3, AMTP). In this pathway, putrescine is metabolized to γ-aminobutyric acid (GABA) via the intermediate γ-aminobutyraldehyde. This metabolic route involves the enzymes putrescine aminotransferase PatA, the γ-aminobutyraldehyde dehydrogenase PatD, the GABA aminotransferase GabT, and the succinic semialdehyde dehydrogenase GabD ending with the formation of succinate that can be further used in the TCA cycle [14,159] (Figure 3, AMTP; Table 1). The activity of the PatA enzyme on cadaverine is reportedly comparable to that towards putrescine.

The acetylation pathway has been described for the utilization of spermidine in *E. coli* and also in *B. subtilis* [206,207,208] (Figure 3, ACP). It occurs via the spermidine acetyltransferase that acetylates spermidine to acetylspermidine using acetyl-CoA [209]. However, the further fate of acetylspermidine in *E. coli* is not yet known (Figure 3, ACP).

Interestingly, the intracellular spermidine concentrations may be also reduced by the glutathionylspermidine synthetase/amidase (GSP synthetase) in *E. coli* and *Haemophilus influenzae*. In the reaction catalyzed by this enzyme, spermidine is ligated with glutathione forming glutathionylspermidine in an ATP-dependent manner [210,211].

##### Polyamine Utilization in *P. aeruginosa*

In *P. aeruginosa* PAO1, the gamma-glutamylation pathway (Figure 3, GGP) includes almost identical metabolic steps as that in *E. coli* [196,212], but is represented by seven *pauA* genes, four *pauB* genes, one *pauC* gene, and two *pauD* genes that are thought to be responsible for polyamine catabolism [195,196] (Table 1). Interestingly, each PauA1-PauA7 enzyme seems to have different substrate specificity towards different mono- and polyamines being involved in the first step of the pathway [195,196].

The aminotransferase pathway (Figure 3, AMTP) of *P. aeruginosa* PAO1 includes a putrescine-pyruvate aminotransferase that generates γ-aminobutyraldehyde and L-alanine [213,214]. The KauB protein that corresponds to PatD from *E. coli* oxidizes in the following step, with γ-aminobutyraldehyde forming GABA, which is further catabolized to succinate by GabT and GabD [14,212] (Figure 3, AMTP; Table 1).

The direct oxidation pathway (Figure 3, DOP) has been investigated in *P. aeruginosa* and *Staphylococcus aureus*. It requires an amine oxidase [195,215,216,217,218,219] (Figure 3, DOP). The spermine/spermidine dehydrogenase pathway has been described in *P. aeruginosa* [195,212], for which the structure of the essential enzyme spermidine dehydrogenase has been reported [216] (Figure 3, SPDP). In *P. aeruginosa* PAO1, the spermidine dehydrogenase (SpdH) can cleave spermidine into 1,3-diaminopropane and γ-aminobutyraldehyde and spermine into spermidine and 3-aminopropanaldehyde. KauB oxidizes 3-aminopropanaldehyde to β-alanine, which is further catabolized to acetyl-CoA [195,212] (Table 1).

##### Polyamine Utilization in *S. coelicolor*

In contrast to Gram-negative bacteria *E. coli* and *P. aeruginosa*, the utilization of polyamines has barely been investigated in Gram-positive bacteria. While some studies report the acetylation of polyamines by *S. aureus* [197], *B. subtilis* [206], and *C. glutamicum* [198], extensive investigations of polyamine catabolism have been reported in *S. coelicolor*. A gamma-glutamylation pathway for polyamine utilization in *S. coelicolor* has been described in a combined in silico and transcriptional analysis [7] (Figure 3, GGP). The first step is catalyzed by GlnA2 and GlnA3 enzymes that are able to glutamylate the polyamines putrescine, spermidine, spermine, and cadaverine [113,115]. Based on in silico and transcriptional studies, the following steps of the pathway were postulated. In the second step, γ-glutamylpolyamines are further reduced by the γ-glutamylpolyamine oxidoreductase (SCO5671). This enzyme is an ortholog of the γ-glutamylpolyamine oxidoreductases PauB1-B4 in *P. aeruginosa* and PuuB in *E. coli* (Table 1). The subsequent step is catalyzed by the dehydrogenases (SCO5657 and SCO5666) that are orthologs of (γ-glutamyl-) γ-aminobutyraldehyde dehydrogenases PuuC and PatD from *E. coli*. The fourth pathway step needs hydrolases (SCO5657, SCO5666, and SCO6961), resulting in production of γ-aminobutyric acid (GABA) or aminovalerate. Subsequently, an ortholog of GabT from *E. coli* the GABA aminotransferase (SCO5676) catalyzes the production of succinate semialdehyde or glutarate semialdehyde (Table 1). Remarkably, it has been shown that the expression of *sco5676* is induced by arginine [70], which is a precursor of putrescine biosynthesis, as well as in the presence of polyamines [113]. Finally, a homolog of the succinic semialdehyde dehydrogenase GabD from *E. coli*, the SCO5679 protein, catalyzes the last step producing glutarate or succinate that feed the tricarboxylic acid (TCA) cycle. RNAseq analysis showed that the expression of *sco5679* was induced by polyamines, supporting the hypothesis that SCO5679 is involved in the last step of the polyamine gamma-glutamylation pathway in *S. coelicolor* [7,115] (Figure 3, GGP; Table 1).

The aminotransferase pathway of *S. coelicolor* includes an aminotransferase. The enzyme SCO5655 is a homolog of the putrescine aminotransferase (PatA) from *E. coli* (Table 1). The RT-PCR analysis demonstrated that the expression of *sco6960*, *sco6961*, and *sco5655* was enhanced in the presence of polyamines [113], indicating their involvement in polyamine assimilation (Figure 3, AMTP).

#### 2.1.5. Regulation of Polyamine Assimilation Genes in Bacteria

Polyamines are involved in the regulation of transcription and translation processes required for bacterial proliferation. Therefore, a strict control of the intercellular polyamine content is of importance. Regulation of the genes from the gamma-glutamylation and aminotransferase pathways has been extensively investigated in *E. coli*. It allows the detection of elevated polyamine concentrations aiming at their subsequent intracellular utilization. It has been demonstrated in *E. coli* that the gene control involves the nitrogen regulatory protein C (NtrC), the nitrogen assimilation control protein (Nac), an alternative sigma factor σ^S^, and the alternative sigma factor for nitrogen-controlled genes σ^54^ [6,14,20]. For instance, the putrescine aminotransferase encoding gene *patA* is regulated by NtrC, σ^S^, and σ^54^ [159,203,220]. Its expression can also be subjected to catabolite repression [214].

The gamma-glutamylation pathway is encoded in the *puuPADRCBE* gene cluster in *E. coli* [180]. Similar to *patA*, this gene cluster contains the σ^S^ dependent promoters [86,221] and the NtrC-σ^54^ dependent promoter [86,222]. The genes of the gamma-glutamylation pathway are regulated by the *puuR* encoded repressor PuuR, which represses the expression of *puu* genes in the *puuAP* and *puuDRCBE* operons [202,223]. It has been demonstrated in *E. coli* that the expression of the *puu* genes can be induced by putrescine as well as through the shift from anaerobic to aerobic conditions. FNR and ArcA recognition sites were described in the *puuA*-*puuD* intergenic region [224].

In *S. coelicolor*, the regulation of polyamine utilization genes involves two regulators. It has been demonstrated that the regulation of the γ-glutamylpolyamine synthetase encoding gene *glnA3* of the gamma-glutamylation pathway is controlled by a specific regulator SCO5656 (EpuRII) [115]. In RT-PCR and EMSA analysis, EpuRII revealed control of several polyamine-associated genes, including *glnA3*, *sco5676*, and *sco5977* (Table 1). Another regulator of polyamine utilization genes is the global regulator of the nitrogen metabolism GlnR. This has been demonstrated to control the transcription of the second γ-glutamylpolyamine synthetase encoding gene *glnA2*. According to EMSA analysis, especially strong binding of *glnA2* to the promoter area was observed for the acetylated version of GlnR [115].

### 2.2. Monoamine Metabolism in Bacteria

#### 2.2.1. Distribution and Role of the Monoamine Ethanolamine

Ethanolamine is a naturally occurring common monoamine. It is a primary alcohol and primary amine that belongs to the class of aliphatic amino alcohols. Ethanolamine is a building block of biomembranes and usually occurs in the form of the second-most-abundant head group for phospholipids—phosphatidylethanolamine [225,226,227]. Phosphatidylethanolamine is a substrate and precursor in several biological pathways and comprises 25–45% of all phospholipids in a cell [228]. Ethanolamine forms choline after methylation and is a nitrogenous base in phospholipids and an essential vitamin [227]. Ethanolamine is a main precursor of phosphoglycerides that are important elements in the structure of choline in cellular membranes [229]. Ethanolamine is an abundant compound in processed food and the intestinal tract content [230,231]. It is prevalent in the gastrointestinal tract environment. Ethanolamine and glycerol occur as a breakdown product after the cleavage of phosphatidylethanolamine by phosphodiesterases [232,233].

The incorporation into cell membranes, the importance for cellular homeostasis, and the biological role of ethanolamine have been reported in a number of bacterial genera, such as *Mycobacterium* spp. [234], *Corynebacterium* spp., *Enterococcus* spp., *Listeria* spp. and *Clostridium* spp. [235,236], *Chromohalobacter salexigens* [237], and *Streptomyces* spp. [114]. Ethanolamine has been observed to be a donor of nitrogen and carbon for gut-associated Gram-negative bacteria such as *Escherichia* spp., and *Salmonella* spp. [235,236]. Since ethanolamine as an alternative nitrogen source favors a competitive advantage for intestinal pathogens, its utilization has been described as a positive virulence factor [236,238].

#### 2.2.2. Ethanolamine Biosynthesis and Uptake in Bacteria

**Figure 4:**
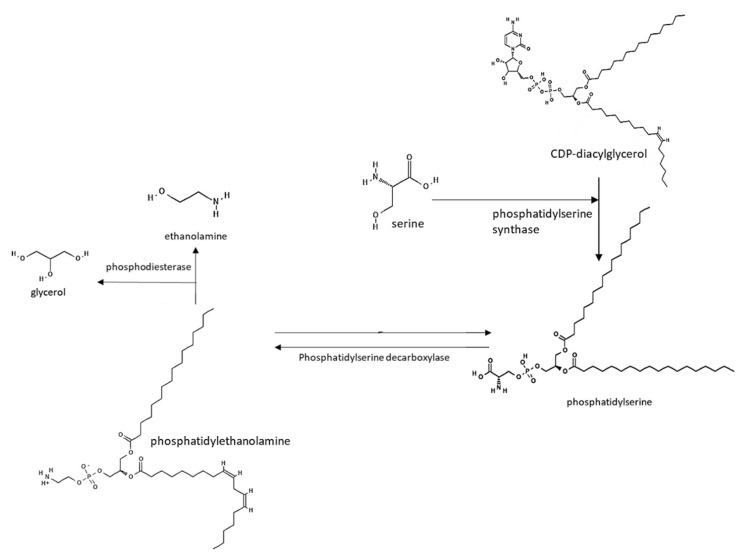
Combined schematic model of ethanolamine and phosphatidylethanolamine biosynthesis in bacteria [229,232,240]. Biosynthetic reactions with the respective substrates, products, and involved enzymes are shown. The bacteria indirectly biosynthesise of ethanolamine from serine via the phosphatidylserine synthase and the phosphatidylserine decarboxylase is depicted. CDP: cytidine diphosphate.

The direct biosynthetic pathway for ethanolamine is the decarboxylation from serine (Figure 4). This metabolic route has been investigated in plants and mammals, but it is barely studied in bacteria. Yeast and bacteria possess a phosphatidylserine decarboxylase and can synthesize phosphatidylserine in a reaction catalyzed by a phosphatidylserine synthase. This enzyme condenses the phosphatidyl moiety of cytidine diphosphate (CDP)-diacylglycerol with serine, resulting in phosphatidylserine [229,239] (Figure 4).

Bacteria can also take up extracellular ethanolamine in order to optimize the metabolism and environment adaptation. The uptake of ethanolamine is possible through the passage into the cell by carrier-mediated transport or diffusion [241]. In *E. coli* and *S. typhimurium*, it can be transported into the cell by the specialized transport protein EutH, which is related to permeases [242]. Most Actinobacteria and Proteobacteria can obtain ethanolamine from the extracellular environment using a transporter encoded by the *eat* gene, which is a functional, non-homologous equivalent to *eutH* from *S. typhimurium* [243].

#### 2.2.3. Ethanolamine Assimilation in Bacteria

**Figure 5:**
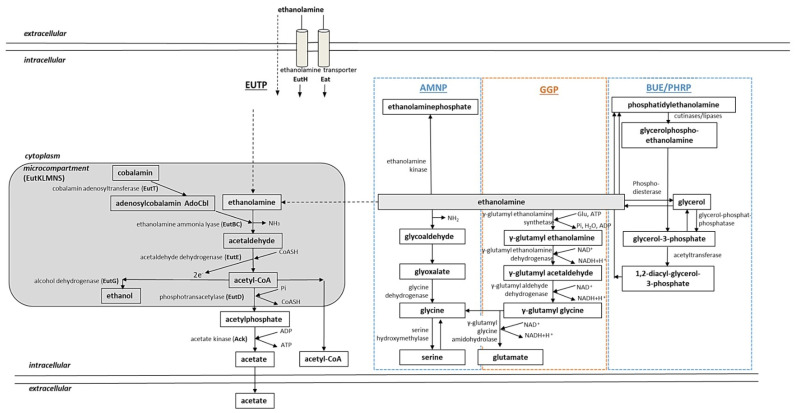
Combined model of bacterial ethanolamine utilization pathways (adapted from [7,114]). Central routes of ethanolamine catabolism with reaction products and involved enzymes, as well as up-to-date polyamine uptake systems known in bacteria, are shown. EUTP: *eut*-pathway; AMNP: amination pathway; GGP, gamma-glutamylation pathway; BUE: biosynthetic utilization of ethanolamine; PHRP: polar head recycling pathway. Dashed arrows represent diffusion. Gray, rounded rectangle represents the bacterial microcompartment. Pathways in black: *E. coli* and *S. typhimurium*; dashed blue box: *Mycobacterium* sp. and *M. tuberculosis*; dashed orange box: *C. salexigens* and *S. coelicolor* [7,234,236,237,243,249,250,251,254,264].

Ethanolamine utilization as a source of carbon and nitrogen has been reported and investigated in Salmonella, Enterococcus, Arthrobacter, Erwinia, Flavobacterium, Klebsiella, Mycobacterium, Pseudomonas, Achromobacter, Corynebacterium, Clostridium, Vibrio and Escherichia [235,243,244,245,246,247,248], Chromohalobacter [237], and Streptomyces [114]. Members of the Enterobacteriaceae family such as E. coli and S. typhimurium, as well as the members of the phylus Firmicutes, possess long eut operons encoding genes for ethanolamine utilization in a eut-pathway (Figure 5, EUTP). These operons have considerable differences in gene content, organization, and regulation [235,243]. Actinobacteria and most Proteobacteria feature short eut operons, which contain the transporter encoding gene eut together with eutBC and sometimes eutR [235,243]. There are species that contain both long and short eut operons: Klebsiella pneumonia and Pseudomonas fluorescens [243].

It has been observed that organisms ranging from the Proteobacteria to Actinobacteria possess the capability for ethanolamine metabolism that does not require *eut* genes. In addition to canonical ethanolamine utilization pathways that involve *eut* genes and the metabolosome, an alternative conversion of ethanolamine has been reported [114,234,237,249,250,251]. The following pathways have been characterized in *E. coli*, *S. typhimurium*, *Mycobacterium* sp. 607, *M. tuberculosis*, *C. salexigens*, and *S. coelicolor*: the *eut* pathway (Figure 5, EUTP), the amination pathway (Figure 5, AMNP), polar head recycling (Figure 5, PHRP), biosynthetic utilization of ethanolamine (Figure 5, BUE), and the gamma-glutamylation pathway (Figure 5, GGP).

##### Ethanolamine Assimilation in *E. coli* and *S. typhimurium*

*E. coli* and *S. typhimurium* possess similar ethanolamine utilization mechanisms involving proteins encoded by *eut* genes [231,246] (Figure 5, EUTP). Ethanolamine utilization involves 17 Eut proteins encoded by genes from the ethanolamine utilization operon *eut* [242,252,253]. Ethanolamine utilization in *E. coli* and *S. typhimurium* takes place in a bacterial microcompartment (BMC) [254], also known as the metabolosome [231,255], which is required to retain acetaldehyde. Microcompartments protect the cell from toxic effects by acetaldehyde and prevent the loss of this volatile C-source [256,257].

Ethanolamine can enter the cell by diffusion or via the ethanolamine transporter EutH. After it reaches the microcompartment, the ethanolamine ammonia lyase EutBC breaks down ethanolamine into ammonia and acetaldehyde [252,258,259]. This process requires a cofactor AdoCbl (adenosylcobalamin), which is generated from cobalamin by a corrinoid cobalamin adenosyltransferase EutT [253]. The ammonia serves as a supply of reduced nitrogen. The acetaldehyde is transformed into acetyl-CoA by an acetaldehyde dehydrogenase EutE [252,260]. Acetyl-CoA is used in the TCA cycle, the glyoxylate cycle, and lipid biosynthesis [243]. Acetyl-CoA is converted into acetylphosphate by a phosphotransacetylase EutD or into ethanol by an alcohol dehydrogenase EutG [242]. Acetylphosphate is converted into acetate by an acetate kinase Ack generating ATP [261,262] (Figure 5, EUTP).

The ethanolamine-specific microcompartment of *S. typhimurium* also contains other structural proteins for microcompartment formation encoded by *eutK*, *eutM*, *eutS*, *eutL*, and *eutN* [231,254]. Other proteins encoded by the *eut* cluster can indirectly influence ethanolamine utilization. Such proteins include a reactivating factor for the ethanolamine ammonia lyase EutA and EutJ (chaperone of EutG and EutE), as well as EutP and EutQ [243].

##### Ethanolamine Assimilation in *S. coelicolor* and *M. tuberculosis*

In *S. coelicolor*, ethanolamine utilization has been shown to occur via gamma-glutamylation [114] (Figure 5, GGP). In the first step of the pathway, ethanolamine is glutamylated by the γ-glutamylethanolamide synthetase GlnA4 (SCO1613). Based on bioinformatical and transcriptional analysis, it was possible to describe the subsequent steps of the pathway: it involves a γ-glutamylethanolamine dehydrogenase SCO1611, a γ-glutamylaldehyde dehydrogenase SCO1612, and a γ-glutamylglycine amidohydrolase SCO1615. The end products of the pathway are glycine and glutamate [114]. These pathway steps have also been proposed for *C. salexigens* [237,251] (Figure 5, GGP; Table 2).

Studies in vivo revealed that *Mycobacterium* sp. are not only able to use host fatty acids from the lipid droplets, but also acquire carbon and nitrogen from phospholipids of the host [217]. The human pathogenic Actinobacteria had to evolve a direct metabolic pathway to strictly control the intracellular ethanolamine due to its potential toxic effect when in excess. Accumulation of ethanolamine might lead to its uncontrolled binding to negatively charged molecules such as DNA or RNA, alkalization of the cellular milieu, and cellular death. It has been demonstrated that *Mycobacterium* sp. including *M. tuberculosis* are able to degrade and recycle their own as well as host phospholipids and use them as nutrients [263]. During the recycling process, phosphatidylethanolamine is cleaved by phospholipases. Subsequently, the lipid polar head (glycerolphosphoethanolamine) is converted to glycerol-3-phosphate and ethanolamine by glycerophosphoryl diester phosphodiesterases (Figure 5, PHRP). The phosphodiesterase is essential in vivo during survival of *M. tuberculosis* in macrophages [264]. Glycerol-3-phosphate can then be channeled for glycolysis, gluconeogenesis, and acylation or broken down to glycerol and phosphate. In *Mycobacterium* sp., ethanolamine can also be transformed into phosphatidylethanolamine (Figure 5, BUE), which occurs in a pathway involving phosphatidylserine synthase with the intermediate phosphatidylserine (as described in the Section 2.2.2). Furthermore, in *Mycobacterium* sp., ethanolamine can be transformed to glycoaldehyde and further to glyoxate, which is converted to glycine by glycine dehydrogenase [234,249,250,265] (Figure 5, AMNP).

#### 2.2.4. Regulation of Ethanolamine Assimilation Genes in Bacteria

Intracellular ethanolamine content can drastically increase as a result of uptake or cell lysis. In order to reduce elevated ethanolamine concentrations and escape the cytotoxic effects, strict control of the ethanolamine utilization genes is essential. The regulation mechanism of ethanolamine-associated genes in bacteria has been investigated in *E. coli*, *S. typhimurium* (the EutR system), and *Enterococcus faecalis* (the EutV-EutW system). In *S. typhimurium*, a EutR regulator positively regulates the transcription of the *eut* operon in the presence of ethanolamine [252,266]. EutR belongs to the AraC family of transcriptional regulators. It binds the promoter of *eut* in the presence of ethanolamine and AdoCbl as well as the promoter of the *eutR* gene, providing a low level of constitutive expression [267,268]. Autoregulation of *eutR* allows the maintenance of induced expression despite competition between EutR and EutBC for AdoCbl, which is required for synthesis of EutBC and for the regulation of the *eut* operon by EutR [243,267].

*E. faecalis* does not have the *eutR* gene. In this organism, ethanolamine utilization is regulated by a two-component system composed of the response regulator EutV and the sensor histidine kinase EutW [245]. It has been demonstrated that *eut* operon regulation can involve other regulators. For example, the expression of the *eut* operon in *S. typhimurium* can also be modulated by the global regulator of invasion genes CsrA, which can lead to increased pathogenicity [266,269]. Furthermore, the expression of the *eut* operon in *E. faecalis* can be influenced by the global transcriptional regulator of gelatinase and serine protease encoding genes Fsr [243,269].

In contrast to *S. typhimurium* and *E. faecalis*, regulation of ethanolamine utilization in Actinobacteria remains less investigated. It has been shown that in *S. coelicolor*, the gene *sco1614* (*epuRI*) encodes a regulator of the γ-glutamylethanolamide synthetase encoding gene *glnA4*. The role of EpuRI as a negative transcriptional regulator of the genes associated with ethanolamine utilization has been proven by transcriptional analysis [114].

## 3. Recent Advances in Drug Development Targeting Bacterial Nitrogen, Mono- and Polyamine Metabolism

The investigation of the crucial role of nitrogen metabolism and specifically polyamine and monoamine metabolism for survival of pathogenic bacteria and human parasites led to the discovery of a number of potential drugs that might inhibit key enzymes. A large group of such compounds include GS inhibitors that have been extensively investigated in connection to the *M. tuberculosis* infection and can be described in two categories. The first group are small, highly polar amino acid analogues that target the conserved amino acid binding site, e.g., two of the most widely used GS inhibitors are methionine sulfoximine (MSO) and phosphinothricin (PPT). The second group are the larger, more hydrophobic heterocycles that compete with ATP targeting the nucleotide-binding site, e.g., purine analogs [98,270]. Furthermore, azaserine, an inhibitor of GOGAT in *M. tuberculosis*, has been identified [271]. GDH has also been described as an imported drug target in *Mycobacterium* spp.

Another group of inhibitors include compounds that target the polyamine biosynthesis (see pathway, Figure 2). Such inhibitors include the D,L-α-difluoromethylornithine (DFMO). It is a fluorinated ornithine analog that targets polyamine biosynthesis by inhibiting the ODC. DFMO is an approved drug, not only for cancer treatment [272] but also to treat trypanosomiasis [273] and *Streptococcus pneumoniae* infections [274]. Other validated ODC inhibitors include the putrescine analogs 3-aminooxy-1-aminopropane (APA) and 1,4-diamino-2-butanone (DAB), the agmatine analog 1-guanidinooxy-3-aminopropane (GAPA), and the spermine analog MDL 27695 (N,N′-bis(3-((phenylmethyl)amino)propyl)-1,7-diaminoheptane) that have been shown to be effective against *Leishmania* spp. [273]. Further inhibitors of polyamine metabolism studied in *Leishmania* spp. are AdoMetDC inhibitors, e.g., 5-(((Z)-4-amino-2-butenyl)methylamino)-5-deoxyadenosine (MDL 73811) and CGP 40215A (a diamidine and bicyclic analog of MGBG), as well as SpdSyn inhibitors, e.g., hypericin [172,273]. Other polyamine analogs include transport inhibitors such as Ant4 analogs identified in a study with *T. cruzi* [275] and AMXT 1501, which has been described as a potent inhibitor of polyamine and capsule biosynthesis in *S. pneumoniae* infections [274]. Polyamine biosynthesis and transport have been identified as drug targets in some bacteria as *S. pneumoniae* and *Salmonella enterica* serovar Typhimurium [276,277].

Remarkably, the development of drugs targeting ethanolamine metabolism is far less investigated. It has been reported that ethanolamine analogs with substitutions in the amino group of one of the methylene hydrogens of ethanolamine effectively inhibit ethanolamine transport in *T. cruzi* [278]. Although polyamine and ethanolamine utilization have been reported to be excellent drug targets in pathogenic bacteria, the development of inhibitor candidates still requires further study.

## 4. Conclusions

Mono- and polyamine metabolism has been investigated in a number of human pathogenic parasites and bacteria as a drug target. The knowledge of these metabolic networks is of medical importance, allowing the development of new drugs based on the validation of key enzymes involved in biosynthesis and utilization of these compounds. Multiple studies in pathogenic parasites including *Leishmania* sp., *Trypanosoma* sp., *Toxoplasma* sp., *Trichomonas* sp., *Cryptosporidium* sp., *Crithidia* sp., and *Leptomonas* sp. [279,280], and bacteria *S. typhimurium*, *B. abortus*, *M. tuberculosis*, *Chlamydia pneumoniae*, *Legionella pneumophila*, *Listeria monocytogenes*, and others [281] prove the crucial role of polyamines for their proliferation [282,283] leading to the validation of specific enzymes as drug targets. The interconnection between the mono-/polyamine biosynthesis, uptake, and assimilation remains crucial to find new drug targets. Since most human pathogens rely not only on polyamine biosynthesis, but also on polyamine detoxification with possible subsequent utilization or efflux in order to proliferate and maintain infection, targeting mono-/polyamine metabolism can extend the options for combating bacterial infections. Interestingly, targeting polyamine metabolism is currently also receiving much attention as a potential anti-cancer treatment [284,285], leading to the occurrence of validated inhibitors.

Investigations of mono- and polyamine metabolism in model bacteria including *E. coli*, *S. typhimurium*, *P. aeruginosa*, and *S. coelicolor* suggest new applications. For example, gamma-glutamylation pathways for polyamine utilization are rather of advantage for bacteria and involve specialized glutamylating enzymes for polyamine detoxification. Such enzymes have been discovered in pathogens *P. aeruginosa* [195] and *S. coelicolor* [113]. These gamma-glutamyl-polyamine/monoamine synthetases modify substrates by adding a glutamyl group in order to ensure the incorporation of these molecules into carbon and/or nitrogen metabolism [7]. Such glutamylation reaction is widespread in nature and might dramatically change chemical features of compounds through the change of stability in solution and by reduction of toxicity, for instance, of gamma-glutamypolyamines/monoamines [286]. Homologs of the glutamylation enzymes GlnA2, GlnA3, and GlnA4 from *S. coelicolor* have been found in other actinobacteria, including human pathogens belonging to *Mycobacterium* spp. and *Rhodococcus* spp. [7]. These enzymes are promising drug targets due to their key relevance in pathogenicity. Almost all actinobacteria, including *M. tuberculosis* causing tuberculosis infection, possess GlnA3 homologues as well as other homologues of polyamine uptake systems and enzymes from the predicted polyamine utilization pathway. Interestingly, the presence of GlnA3 (Rv1878) and some homologues involved in polyamine uptake (Rv1877) and utilization steps GabT (GABA transaminase, Rv2555) were reported in a guinea pig model of tuberculosis, where the bacterial proteome during early and chronic stages of this disease in vivo was investigated [287]. This report provided evidence that homologues involved in the polyamine utilization in *S. coelicolor* are necessary for *M. tuberculosis* surviving during tuberculosis infection.

Since there is an urgent need to find new anti-bacterial drugs with new modes of action that would be efficient on bacterial infections and shorten the treatment duration while avoiding relapses and the emergence of resistances as well as improving compliance, new strategies for the treatment of multidrug-resistant (MDR) infections are of particular concern. Whereas several antibiotics are effective in treating bacterial infections, these drugs target a small number of essential functions in the cell. Therefore, investigation of the pathways for mono-/polyamine metabolism that are required for bacterial growth, survival, and pathogenicity would provide new targets for the rational design of more effective agents that could be active against multidrug resistant strains.

## Figures and Tables

**Table 1:** List of proteins involved in polyamine utilization in *E. coli*, *P. aeruginosa*, and *S. coelicolor* [113,115].

Annotated Function	Homologue in *E. coli*	Homologue in *P. aeruginosa*	Homologue in *S. coelicolor*
Polyamine ABC transporter ATP-binding protein PotA-like	PotA (b1126)/YdcT (b1441)	PAO603/PAO326	SCO3453
Polyamine ABC transporter ATP-binding protein PotC-like	PotC (b1124)/YdcV (b1443)	PAO324/PotC (PA3609)	SCO3454
Polyamine ABC transporter protein	PotB (b1125)/YdcU (b1442)	PotB (PA0205)/PA3252	SCO3455
Polyamine ABC transporter protein—substrate binding protein	YnjB (b1754)	PA0203	SCO3456
Amino acid/polyamine permease	PuuP (b1296)/PlaP (b2014)	PA5510	SCO5057
Lysine/ornithine decarboxylase-like enzyme	-	-	SCO5651
Pyruvate-polyamine aminotransferase	PatA (b3073)	SpuC (PA0299)	SCO5655
Lrp/AsnC family transcriptional regulator	-	-	SCO5656
γ-aminobutyraldehyde or γ-glutamyl-γ-amino-butyraldehyde dehydrogenase	PatD (b1444)/PuuC (b1300)	BetB (PA5373)/PAO219	SCO5657
Polyamine-binding lipoprotein	PotF (b0854)	SpuD (PA0300)	SCO5658
γ-aminobutyraldehyde dehydrogenase or 4-guanidino-butyraldehyde dehydrogenase	PatD (b1444) PuuC (b1300)	PauC/KauB (PA5312)	SCO5666
Polyamine ABC transporter substrate-binding protein	PotF (b0854)	SpuE (PA0301)	SCO5667
Polyamine ABC transporter substrate-binding protein	PotG (b0855)	SpuF (PA0302)	SCO5668
Polyamine ABC-transporter integral membrane protein	PotH (b0856)	SpuG (PA0303)	SCO5669
Polyamine ABC-transporter integral membrane protein	PotI (b0857)	SpuH (PA0304)	SCO5670
γ-glutamyl-polyamine oxidoreductase	PuuB (b1301)	PauB3 (PA2776)	SCO5671
γ-aminobutyrate aminotransferase gabT-like or puuE-like	GabT (b2662)/PuuE (b1302)	GabT (PA266)	SCO5676
Succinate-semialdehyde dehydrogenase gabD-like	GabD (b2661)	GabD (PA0265)	SCO5679
Amino acids/polyamine permease	PuuP (b1296)	PA5510/PAO322	SCO5977
Hydrolase	-	-	SCO6960
Amidohydrolase	-	-	SCO6961
γ-glutamyl-polyamine synthetase	PuuA (b1297)	PauA7 (PA5508)/SpuI (PA0296)	SCO6962

**Table 2:** Combined list of enzymes involved in ethanolamine utilization in *C. salexigens* and *S. coelicolor* [114,237].

Annotated Function	Orthologues in *C. salexigens* (Protein Family)	Orthologues in *S. coelicolor*
γ-glutamyl ethanolamine synthetase/ethanolamine γ-glutamylase	PF00120	SCO1613
γ-glutamyl ethanolamine dehydrogenase/iron-dependent dehydrogenase	PF00465	SCO1611
γ-glutamyl aldehyde dehydrogenase	PF00171	SCO1612
γ-glutamyl glycine amidohydrolase /formylglutamate amidohydrolase	PF05013	SCO1615

## Data Availability

Data are involved in this study.
